# Computer Education and Third Age Universities: A Systematic Review

**DOI:** 10.3390/ijerph18147390

**Published:** 2021-07-10

**Authors:** Sónia Rolland Sobral, Margarida Sobral

**Affiliations:** 1 Universidade Portucalense, 4200 Porto, Portugal; 2 Psychogeriatrics Service, Hospital Magalhães Lemos, 4149 Porto, Portugal; margaridasobral@hmlemos.min-saude.pt; 3 CINTESIS (Center for Health Technology and Services Research), 4200 Porto, Portugal

**Keywords:** elderly, U3A, technology skills, ICT, computer class

## Abstract

An aging population and a digital society are realities. There is a need to equip older people with knowledge and computer skills so that they can participate in society, without feeling excluded or being marginalized. Third age universities are organizations around the world that specialize in teaching and learning for senior students in a more informal and more integrated way than other educational institutions. The objective of this study was to identify the existing quality publications that deal with the subject of computer education at senior universities. The SCOPUS and Web of Science databases were used, and 18 records were found according to the adopted criteria. It was found that these articles, depending on their focus, can be divided into four groups: educators, organizations/directors, students, and conceptual/review papers. Through these articles, it was possible to draw a picture of what older people’s computer learning is like, what barriers exist for students to not be able to attend these classes, as well as tips on how courses should be organized and the pedagogical methodologies that must be adopted. It is intended that this article is used as a good tool for people who work in teaching information technology to the elderly, and especially for course directors who intend to create or reformulate courses of this type for this specific age group.

## 1. Introduction

Internet use by the elderly has increased. In 2019, 58% of people aged between 55 and 74 used the Internet frequently, compared to only 30% in 2010. However, these percentages are still far from the 95% of the 16–24 age group (people in Organization for Economic Co-operation, and Development, OECD) [1]. These numbers refer us to the “no one will be left behind” promise of the 2030 Agenda for Sustainable Development, which aims to eradicate poverty in all its forms and achieve global sustainable development, namely ensuring healthy lives and well-being at all ages (third Sustainable Development Goal; SDG 3) [2]. Population aging takes place all over the world. In 1990, 6% of the population was aged 65 and over; in 2019, 9% of it was, and in 2050, it is projected to be 16% [3]. At the beginning of the century, the issue of old age and development was already recognized as a priority. “Older persons must be full participants in the development process and share in its benefits. No individual should be denied the opportunity to benefit from development” [4]. Wide public access to computers and the Internet, with no or minimal cost, in public places such as government offices, community centers, and libraries is on the World Health Organization (WHO) checklist of essential features of age-friendly cities [5].

Increasing older adults’ willingness to use technologies will fill the digital gap caused by aging and increase the prevalence of emerging technology products, which can help facilitate independent living for older adults [6]. Internet use reduces isolation and/or exclusion, consequently increasing the quality of life, especially for the elderly [7]. The exclusion and marginalization of older people can be reduced with the use of technology [8], increasing digital literacy—the skills required to achieve digital competence, the confident and critical use of information and communications technology (ICT) for work, leisure, learning, and communication [9]—and decreasing the digital divide in the access to and use of technology. The elderly are not digital natives but digital emigrants; in other words, they must “acculturate to the new world to which they emigrated” [10]. Many people had no contact with ICT during their professional life, which makes it difficult to use computers in old age. Some studies have established a relationship between the highest educational level and the best use of technology [11], and the encouragement by family and friends is a strong predictor for Internet use [12].

Using the Internet to communicate with family and friends is the most common activity performed by older adults, who use computers primarily for email and social media [13]. Elderly people are not a homogeneous group, nor are they homogeneous in their practices on the Internet, and some studies include different clusters for their Internet browsing experience that seem indifferent to social and health-related variables [14]. Due to the COVID-19 pandemic and the resulting isolation of people, the use of information technologies by the elderly has intensified for some videoconferencing apps and services, such as Zoom, for both business and social activities [15]. Some innovations have been made, such as Komp [16], a one-button computer made specifically for isolated individuals who have little or no experience using smartphones, computers, or tablets, and the Nextdoor app [17], used to connect with the neighborhood. The COVID-19 pandemic and its consequences have enhanced the importance of Gerontechnology, a term that the International Society for Gerontechnology defines as designing technology and the environment for an independent life and the social participation of elderly people with good health, comfort, and safety [18], an interdisciplinary field combining gerontology and technology [19]. Due to the characteristics of the older population [20], there is a need for the design of technological tools to became elderly-friendly tools, such as the obvious increase in font size [21], a sharp contrast between the background and font color [22], and large tap areas [23]. In this sense, and as a cause of an aging population, many studies have been published; for example, research on dementia is developing exponentially following the evolution of the widespread use of computer science [24]. 

The involvement of older people in education can potentially have positive effects on cognition and psychological well-being, as well as preventing social isolation [25]. The benefits of active aging are known: seniors with high participation in leisure activities may benefit from a slower cognitive and functional decline [26]. The participation of older adult students in educational activities contributes to successful aging (activity, adaptability, and attitude) and to connectivity factors (spiritual, social, personal, and future) [27]. The role of senior universities is undeniable, generally open to all in the third age, which is defined not by a certain age, but by a period of life in which full-time employment has ended [28], recognizing the fact that older people are a diverse group with different needs, abilities, backgrounds, and experiences [29]. If at first, one of the objectives of seniors’ universities was personal contact, that is, in-person, face-to-face contact, to promote student interaction, at this moment, there are several opportunities in distance education for the elderly to increase the “presence” of people who, for physical or geographical reasons, were previously unable to participate [30,31]. The presence of older adults in Massive Open Online Courses (MOOCs) is reported in the literature—for all, not for a specific age group [32,33]. Thus, pedagogical strategies must be adjusted for the audience [34]; the way seniors learn to deal with technology is different from the way younger people learn [35].

From the above, the importance of third age universities in the teaching and learning of information and communication technologies seems clear to us. The aim of this study was to analyze the production of literature of proven quality on the teaching of ICT to older people in senior universities. Using PIO (Population/Problem/Patient; Intervention/Issue; Outcome), a version of PICO (Population/Problem/Patient; Intervention/Issue; Comparison; Outcome) [36] without “C” (comparison), we used the following formulation: the Population is people more than 65 years old, the Intervention is university of the third age, and the Outcome is computer education. This article is divided into the methodology, the results, a discussion of the results, and conclusions.

## 2. Methods

A research review is “a systematic, explicit, and reproducible method for identifying, evaluating, and interpreting the existing body of recorded work produced by researchers, scholars, and practitioners” [37]. Arlene Fink divides research literature reviews into seven steps: research questions, bibliographic databases, choosing search terms, applying practical screening criteria, applying methodological screening criteria, doing the review, and synthesizing the results. This systematic review was conducted and reported according to the quality standards described in “Preferred Reporting Items for Systematic Reviews and Meta-Analyses: The PRISMA Statement” [38], namely their Table 1. The checklist of items to include when reporting a systematic review or meta-analysis includes eligibility criteria, information sources, search strategy, and selection process.

### 2.1. Eligibility Criteria

Our formal study began with the definition of the criteria for inclusion and exclusion of existing studies, as well as the method of grouping them. There are several specialized frameworks that help with investigations, such as SPIDER (Sample, Phenomenon of interest, Design, Evaluation, Research type) [39] and SPICE (Setting, Perspective, Intervention, Comparison, Evaluation) [40], for qualitative research questions. There is also CLIP (Client, Location, Improvement, Professionals) and ECLIPSE (Expectation, Client, Location, Impact, Professionals, Services) for health policy and management information [41]. PICO [36] is the most widely used model for formulating clinical questions [42] for quantitative systematic reviews. There are several models created based on PICO, for example, PICOT (Time) [43], PICOTT (Type of question and Type of study design) [44], PICOS (Study design) [45], PIPOH (changing comparators by Professionals and Health care setting) [46], PECORD (Results and Duration) [47] or PESICO (Person, Environments, Stakeholders, Intervention, Comparison, and Outcomes) [48].

Concretely, how does the intervention, in the specified population, produce the expected outcomes? In our study, the population is people more than 65 years old, the intervention is university/education, and the outcome is computer education. In this case, the comparison does not make sense. Thus, our framework was “PIO” (Population; Intervention/Issue; Outcome). Jill Jesson et al. [49] suggest some tips to find the keywords for searching select database words from your research statement or research question and identifying similar and related words and synonyms. The population and intervention can be put into a sentence or phrase, for example, “senior university that can have several denominations”. Through our research, we found “older adult education”, “Universities of the Third Age”, “Third Age university”, “lifelong learning institute”, and the plural forms, “third age universities” and “senior universities”. We also replaced “computer education” with similar keywords, including “computer class”, “digital skills”, “ICT”, and “information and communication technologies”.

As for the characteristics used as eligibility criteria (for example, years considered, language, status of publication), we decided not to limit the study to a language (usually English) because, in this way, we were able to make our study broader. We could eventually find records with articles written in Spanish, Portuguese, or any other language. We considered all articles from the year 2000 (inclusive) until 2 March 2021 and all types of articles.

### 2.2. Information Sources

We decided to use the two world-leading databases, the Web of Science (WoS) and SCOPUS. It has been verified that these two databases can include many others [50,51]. For research with the Web of Science, we used the option to include all databases, which includes WOS, CCC, KJD, MEDLINE, RSCI, and SCIELO. Google scholar, despite having many more records than the chosen databases [52], was not an option because it includes many books, theses, and publications that may not have been peer-reviewed.

### 2.3. Search Strategy

The full electronic search strategy for SCOPUS was TITLE-ABS-KEY (“older adults education” OR “senior university” OR “senior universities” OR “Universities of the Third Age” OR “Third Age university” OR “third age universities” OR “lifelong learning institute”) AND ALL (“computer class” OR “digital skills” OR “ICT” OR “information and communication technologies” OR “computer education”) AND PUBYEAR > 1999.

The full electronic search strategy for the Web of Science (WOS, CCC, KJD, MEDLINE, RSCI, SCIELO) was TS = (“older adults education” OR “senior university” OR “senior universities” OR “Universities of the Third Age” OR “Third Age university” OR “third age universities” OR “lifelong learning institute”) AND TS = (“computer class” OR “digital skills” OR “ICT” OR “information and communication technologies” OR “computer education”) AND PY = (2000–2021).

### 2.4. Selection Process

Some research result records were found that were not related to the present study, such as senior students at university (in the sense of older, end of course students), which is different from people studying at senior universities. Both authors worked with the databases, double-checking the records and the conditions of eligibility and ineligibility.

## 3. Results

**Figure 1:**
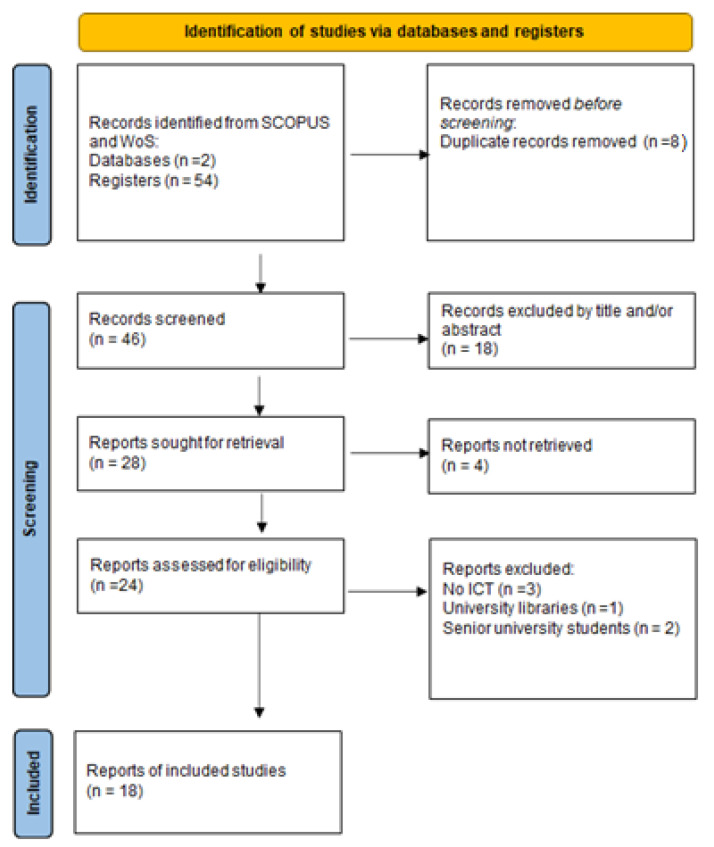
Study selection flow diagram (adapted from the PRISMA diagram) [53].

The literature search identified a total of 46 unique records after removing the duplicate records retrieved by SCOPUS (n = 40) and WoS (n = 14). A total of 18 records were excluded by title and/or abstract, 4 records because we could not access the entire text, and 6 for other reasons (no computer class or no third age university). Figure 1 shows the study selection flow diagram (adapted from the PRISMA diagram) [53].

The results were divided by bibliometrics and content analysis into four categories: educators, organization, students, and conceptual/review papers.

### 3.1. Bibliometric Analysis

Bibliometric analysis has been widely used to analyze published literature in a particular field. This technique is a useful tool to assess the trend of research activities over time. A bibliometric analysis of international papers is a way to provide a valuable reference for future research [54].

The articles found in the research were published between 2010 and 2020, with the years 2016 and 2018 having the highest number of publications (n = 4). A total of 66% of the publications are journal articles, with the remaining 33% being conference proceedings. There are 14 different sources; 12 of them have one publication and 2 of them have three. These 2 publications are a journal and a conference proceeding. The journal is *Gerontology and Geriatrics Education*, with an H-Index 24 and a quartile of Q3 in the sub-area of medicine, Geriatrics, and Gerontology, Q2 in the sub-areas of social sciences and education at Scimago [55], indexed in the Education and Educational Research category in the Emerging Sources Citation Index (ESCI), and in Education|Geriatrics in MEDLINE^®^, PubMed^®^, and Index Medicus^®^ [56]. The conference is CISTI (Iberian Conference on Information Systems and Technologies), with an H-Index of 15 in Computer science, Computer Networks and Communications and Information Systems at Scimago, and in the Web of Science categories of Computer Science and Information Systems.

**Figure 2:**
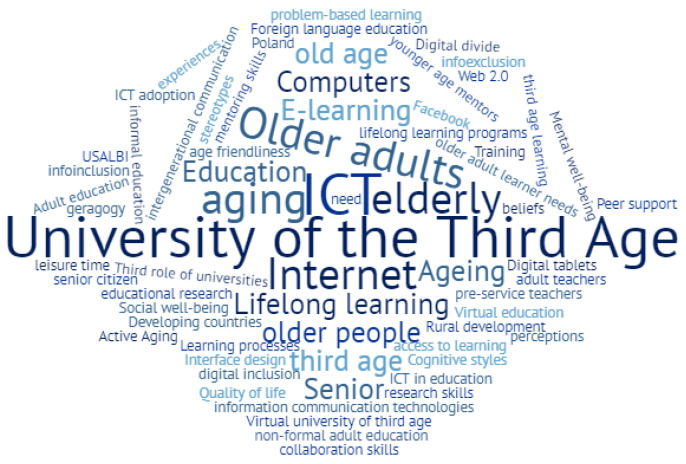
Keyword cloud.

There were 67 keywords provided by the authors. “ICT” is the one that appears most often (in 5 publications), followed by “older adults” and “University of the Third Age” appearing 4 times each. Figure 2 shows a word cloud created with the CloudWords.com website [57].

The average number of authors is 3.28, with most of them being written by three authors, and articles being written by 1 to 9 co-authors. There were 55 different authors. Henrique Gil from ESE–Polytechnic Institute of Castelo Branco, Portugal, is the co-author of three works. There is a cluster of four authors (R. Jack Hansen, Craig A. Talmage, Steven P. Thaxton, and Richard C. Knopf) with the National Resource Center for Osher Lifelong Learning Institutes, Hobart and William Smith Colleges, and Arizona State University, United States. A total of 11% of the articles were written by co-authors from different affiliate countries (i.e., Ukraine and Poland; Finland and Japan). There are three articles with affiliated authors in Portugal and another three with affiliations in the United States.

### 3.2. Content Analysis

We divided the types of approach into four different categories: students (10), educators (3), organizers/directors (1), and conceptual/review (3). One of the articles addresses the first three types simultaneously. The objectives of the studies are diverse and are listed in Table 1. (E)ducator, (O)rganization, (S)tudents and (C)onceptual/Review, aim/objective.

#### 3.2.1. Educators

Four of the articles focus on the mentor position. Usually, the mentor’s task is voluntary, and in two of the articles, it is part of the curricular units for university students. A total of 69 students in the first year of the master’s program for teachers in training enrolled in the optional special course focusing on adult education [62], and 18 graduate students in their 20 years old in the Department of Communication and Media Studies chose the intergenerational course as a junior-level, elective course in their main program [69]. The other two studies were composed of 8 educators of older adults (at an activity center, a public library, a nursing home, an non-governmental organization (NGO), and a University of the Third Age) [70] and 5 ICT Teachers at a senior university [68].

In one of the publications [62], a self-assessment was requested before and after mentoring, about their skills in collaboration, mentoring, and research. Previously, 93% of young people confessed to having difficulty in communicating with seniors, although 95% claimed to have been previously involved in teaching older people how to use digital technologies. Prior to the experience, 43% and 32% self-assessed themselves at level 2 (out of 5) for collaboration and research skills, respectively. At the end of the experience, 41%, 36%, and 54% responded to be at level 3 (out of 5) for collaboration, research, and mentoring skills, respectively. The results suggest the development of all three skills in the pre-service teachers because they realized that their performance was important to improve the quality of life of older citizens in the modern information society.

Another article [69] reports that many of the mentors were anxious about their ability to mentor older adults. During the teaching program, each mentor kept a journal of their experiences. There were big changes in what was written throughout the course in the mentors’ journals. In the end, talking comfortably with another person started as a tool for teaching content and ended as a significant result of the interaction. Both generations got comfortable with each other, liked each other, and changed stereotypes. Graduate students seemed to change their ideas about older people and seemed to change the perception of what can happen in the educational experience.

In another experience [70], and after interviews with mentors, it was found that teachers need content and methodological support, and they expect the promotion of the idea of lifelong education (including the development of digital literacy) in society.

#### 3.2.2. Organizers/Directors

For another paper, semi-structured interviews were conducted with 11 people who organize study processes at the University of the Third Age [74]. Through the interviews, it was noticed that the application of ICT in the organization of studies at the University of the Third Age depends on the individuals who organize the studies, the institution’s material resources, the specificity of the faculty, and the needs of the students.

#### 3.2.3. Students

Table 2 summarizes the articles focusing on the students, categorized by reference, the number of people involved in the study, methodology, demographics, and other results. It appears that the numbers of the samples are varied, and most studies used a questionnaire. Only one of the articles [59] has a very different demographic from the others, either in terms of gender or in terms of schooling level.

Two student-focused articles used data from the same questionnaire completed by students at the Osher Lifelong Learning Institute (OLLI) in the United States [63,64]. Demographic characteristics are seen for 2014 and 2016. Comparisons were made with the country’s demographics, and the two years studied were compared. There were attempts to find answers to some questions, including why there are fewer men than women, why there are so many white people, why are there so many people with at least a bachelor’s degree, why is “lack of time” a barrier to attending classes, to what degree are cost and transportation barriers, and to what degree do physical limitations prevent participation.

Another study [58] presents the demographic data from the survey “Continuing Education Benefits of the Open University of the Third Age (UnATI), Brazil”. A 5-point scale was used for beliefs about aging, with measures of 2.79 for cognition, 2.83 for agency, 2.78 for interpersonal relationships, and 2.75 for persona. The study associated notions of a good old age with physical health, good personal relationships, expectations for the future, and satisfaction with life.

The aim of another study [60] was to assess the effects of adapting learning content to cognitive styles on learning outcomes in a sample of older adults and to assess the mediating role of metacognition and self-regulated learning and learning strategies between intrinsic motivation and learning outcomes using path analysis with observed variables. A sample of 106 (94 final) older adults attending the University of the Third Age (U3A) in Italy was divided into two groups based on the learning approach (face-to-face vs. online). The experimental procedure consisted of answering questionnaires (AMOS Cognitive Style Questionnaire, Intrinsic Motivation Scale (IMS), Metacognition and Self-Regulated Learning Scale (MeSRLS), and the Learning Strategies Scale (LSS)). The presentation of the learning units for e-learners was tailored to their cognitive styles, whereas face-to-face learners received the same units without adaptation. Final examinations verified the achievement of the learning outcomes. The conclusions of this study were that learning processes are facilitated by encouraging older adults to engage and persist in learning activities.

The aim of another article [71] was to outline the pedagogical conditions for the successful use of modern ICT in teaching foreign languages to “third age” students, obtained through the implementation of the “IDEA—an open world of information technologies project” by the public youth organization Vida Nova, with the support of Microsoft by the representative office of PH International in Ukraine. There is a proposal of four modules: the basic notions of how to work with a computer (hardware, texts, spreadsheets, presentations, and Internet) (24 h), digital lifestyle (mobile phones, digital cameras, tablets) (10 h), security and confidentiality (Internet risks) (6 h), and personal productivity apps for remote communication (12 h).

The main objective of Pascoa and Gil [68] was to identify the sociocultural factors that influence and condition the option for learning ICT, as well as to know the impacts of this learning on well-being (mental and social) throughout the aging process. In this article, the following objectives were outlined: to characterize the 50+ population with learning in ICT and without learning in ICT from the point of view of sociodemographic variables (age, gender, marital status, education, profession, income); to learn the opinions of the 50+ population regarding ICT learning; to learn the digital skills of the 50+ population who have already been trained in ICT and its applicability in everyday life. The findings of sociocultural factors were needed for communication and combating isolation. The most evident impacts on “Mental Well-being” and “Social Well-being” shape the exercise of memory and intellectual skills, participation and inclusion in the digital society, and the reduction of loneliness. Recommendations were made for teaching methodologies for the elderly, such as small classes and rooms with good lighting and air conditioning. In ergonomic terms, pay attention to the size of the monitor and its lighting, and ensure the keyboard and mouse have a design specially adapted to the mobility of the elderly. Use a good word processor and ensure the internet pages have larger icons and that the life experience of the elderly is used in their learning, respecting the rhythms of each person, starting from contextualized situations, following the teaching in stages, and providing pauses for conversations.

One article [72] aimed to examine the barriers that affect the level of educational participation of older people in China. Using a focus group methodology, five focus groups were assigned based on gender and (in)activity rate in Xi ‘an, China. The focus groups were carried out to identify individual learning experiences, including motivation, learning preferences, and, above all, barriers to participation for seniors who have already participated in U3As and others who have not. Three of the groups included people who had already attended courses at U3As, while the remaining two groups included people who had not attended those courses. The question that was asked to the students was how to break down the dispositional barriers, including the situational, informational, institutional, and physical. The results suggest that they should be implemented with specific training programs, flexible training schedules, and the recognition of their learning needs.

One paper [73] started with a study in which elderly people were helped with using their tablets, and it listed the implications of the interface design that were observed. It was observed that the monthly average of students increased from 11 to 15, but 63% of participants dropped out right at the beginning. It was perceived with this study that the teaching activity must include gestures with the fingers, a distribution of attention, and several paths towards the same goals. It was realized that beginners need individual teaching before group lessons. It was possible to reduce the distraction by bringing together homogeneous groups (separating iPad and Android users) and helping memory through a printed brochure. Group cohesion was reinforced through discussions and the posting of handouts on the group’s web page so that absentees could stay informed. Tablets are appealing to people with limited computer skills, but older people were nervous to explore on their own and commented in a self-deprecating way about their ability to master this technology.

Another study [59] looked at the effects of education on ICT adoption, focusing on the impact of older adult education. It was found that, in addition to the positive effect of formal education, elderly participants in U3A courses showed greater use of ICT. Specifically, ICT adoption has increased by 18.5% for high school graduates and up to 39% for those who have completed an elementary school diploma, so older adults who have achieved a low level of formal education in the past have not only improved their human and social capital by integrating their current knowledge and preserving their cognitive activity, but they could also fill the gap in ICT skills.

Another study [67] aimed to understand the role of Facebook in promoting active aging. The results revealed that Facebook is a digital social network extended to all ages, promoting socialization, fighting isolation, and contributing to lifelong learning. In other words, it promotes more active aging, especially for those with reduced mobility. The teaching–learning model included theoretical knowledge of software and hardware, notions of Windows and the Internet environment, organization procedures, treatment, and the storage of information in memory supports. In practical classes, students performed Google searches, created an email, and used Messenger and YouTube.

Another article [75] reports several observations. On the computer, the elderly look for information about spheres and events of interest, then the news of the day, and information about trips or schedules. They play PC games infrequently but discover games successively. The article emphasizes the idea of heterogeneity, and its dexterity depends on many factors, such as the ownership of a computer, the courage to work on it on one’s own, or the impatience to wait. It concludes that education must become fun to be effective.

#### 3.2.4. Conceptual/Review

In one of the conceptual articles [61], the issue of the info-exclusion of citizens aged 50+ is addressed, along with its consequences for this group of citizens that prevent them from exercising adequate citizenship and consequent social inclusion. This article reports the study at a senior university in Portugal, where, in the first phase, classes were formed and constituted according to the order of enrolment of their students, with the distribution of students being done in a completely random way. In this way, the subjects of the discipline were taught equally to all students, regardless of their level of training or previous knowledge in this area. However, and given the enormous heterogeneity and disparity of knowledge that existed in the students who made up the different “Computer Science” classes, students began to be divided and stratified into three different levels of knowledge. In addition to this computer science unit, a new course on “Digital Citizenship” was created to complement and even serve as a motivation for attending the course on “Computer Science”. The subject “Digital Citizenship” does not involve the use of any type of digital resource and is intended to create a space for discussion and reflection regarding the framing of ICT in the daily routines of older citizens. It is a discipline where, by way of example, concepts such as “Bluetooth”, and “Wi-Fi” are clarified and where a discussion is also promoted regarding their practical implementation.

Another work [65] aimed to propose e-learning modalities and describes the economic benefits of virtual education using an e-learning platform already in operation at a senior university. Bandwidth problems are especially reported in rural regions, as are low computer skills, which are two obstacles to the effectiveness of e-learning at U3As. Geographical advantages are presented for distance places where there are no senior universities. Despite the challenges, good prospects for the virtual U3A are described.

The only review article in the database [66] features a Web of Science search with the keywords (“information communication technology” OR “computer” OR “internet”) AND (“education” OR “training”) AND (“elderly” OR “old people”), resulting in 16 articles. The framework was divided into physical limitations, cost barriers, and low self-confidence. There are also examples of training elderly people in ICT, including teaching content, organizations, methods, and training the trainers, ending with recommendations for developed countries.

## 4. Discussion

The studies that we have in our database and whose focus is the educator were prepared based on students in a curricular unit who practice teaching older people in an Internet-oriented course at a senior university. It was noticed that before starting to teach, young people have many fears and preconceived ideas about the elderly, but this experience teaches them to communicate and realize that they are important to the elderly (their students). There is also a generational issue here. It is clear, then, that it is necessary to create prior knowledge and skills for the young mentors so that they can start their tutoring without so much prejudice and anxiety.

The set of articles focused on the student has different and diversified methodologies. Almost everyone used a questionnaire to obtain the demographic data of people who attended ICT courses at senior universities; other articles used observation, and others used semi-structured interviews. Known difficulties associated with aging were reported, such as anxiety, depression, and sometimes a lack of interest in learning. There is a very common profile of a senior university student: female, married, 70–74 years old, not working, and with at least a bachelor‘s degree level of education. The methodology applied in these classes is very different from other classes with an audience of a different age group. When students do not know absolutely anything beforehand, individual training should be given and only later, in a group, as there is a huge fear of looking ridiculous for not knowing how to use computer tools. These students like to use their life experience as a motto for learning. There should be a break to talk and socialize, and classes should be fun. There is a very high probability of abandonment and there are barriers that are reported, including financial ones and a lack of time (although only a small proportion of the students is still working).

There is a need to create a kind of curriculum with learning objectives and specific learning contents. Thus, implementing this or not, ICT teaching–learning in a U3A does not depend on several factors, including who is responsible for the institution and their skills (or lack of them) with technology.

## 5. Conclusions

The intention of this article was to assess what has been investigated about computer courses and senior universities. In the 18 articles analyzed, there were four types of focus: educator, organization, student, and conceptual review. Most articles focused on the senior student.

With the set of articles, we were able to gauge the demographics of computer science students in senior universities, and we tried to understand what their fears and barriers are that prevent them from learning. The characteristics common to most elderly people were also analyzed. It must be understood that older people are not a homogeneous group, and that everyone has their own characteristics, namely in cognitive and physical terms, as well as in terms of past work and professional experience. The motivations of the elderly are diverse, but they are mainly linked to the need to communicate with friends and family, and at the same time, to feel active and not excluded. It is important to address the general characteristics of this age group (such as less fast, anxious, fear of looking ridiculous) and change teaching strategies according to the group. It is also important to create small classes but with different levels of learning to meet the needs of students. These courses are informal, so learning should be fun, and some breaks should be taken to socialize. Learning for this age group is based a lot on the personal experiences of each student.

Mentors are usually volunteers and without great pedagogical knowledge in this specific age group. It is important that there is teacher/instructor training so that the experience is not frustrating for either the teachers or the students. The question of learning objectives was not very much addressed in the set of articles analyzed, and apparently, it is a result of the competence of the course director. It would be important to create guidelines or curriculum recommendations for various levels of learning to serve as precise guidelines for senior universities.

For future work, the authors of this paper intend to create a set of guidelines, in relational terms, in pedagogical terms, and in terms of learning objectives, that can be consulted and replicated by technology teaching–learning agents at senior universities.

## Figures and Tables

**Table 1:** References, country, focus, and aim of the 18 records.

Author, Year, Reference	Country	E	O	S	C	Aim
Camargo et al., 2018 [58]	Brazil			X		To identify the correlations between beliefs, perceptions, and concepts about old age and the personal experience of aging among the elderly who attend a public university for seniors
Cattaneo et al., 2016 [59]	Italy			X		To analyze the effects of education on Information and Communications Technology (ICT) adoption by focusing on the impact of older adults’ education
de Palo et al., 2018 [60]	Italy			X		To assess the effectiveness of the e-learning content that has been adapted to the cognitive styles of a sample of older adults
Gil et al., 2016 [61]	Portugal				X	To approach the global aging process, with particular emphasis on the EU-27 (27 European Union countries) and Portugal, as well as the issue of info-exclusion of citizens aged 50+ and its consequences for this group of citizens that prevent them from exercising adequate citizenship and consequent social inclusion
Grynova et al., 2020 [62]	Ukraine, Poland	X				To substantiate the educational strategies of collaboration, research, and mentoring skills development in pre-service teachers facilitating older adults’ learning of ICT with the use of problem-based practice-oriented adult learning activities
Hansen et al., 2019 [63]	USA			X		To describe the demographic characteristics of Osher Lifelong Learning Institute (OLLI) students, changes from 2014 to 2016, to make comparisons with national trends, to investigate the barriers to participation identified by older students participating in OLLIs, to consider studies that have addressed such obstacles for underrepresented groups
Hansen et al., 2020 [64]	USA			X		To assess whether technology-based instruction (TBI) can enhance or complement the in-person lifelong learning experience
Jarolímek et al., 2010 [65]	Czech Republic				X	To propose and verify new modalities of distance education carried out through multimedia tools
Nguyen et al., 2014 [66]	Finland, Japan				X	To review the difficulties that older people have in using computers and the lessons learned in ICT training
Páscoa et al., 2012 [67]	Portugal			X		To understand the role of Facebook in promoting active aging
Pascoa et al., 2017 [68]	Portugal	X	X	X		To identify the sociocultural factors that influence and condition the option of learning ICT and knowing the impacts of this learning on well-being (mental and social) throughout the aging process
Shedletsky, 2012 [69]	USA	X				To explore the perceptions of the undergraduates as they interact with older adults
Tomczyk et al., 2020 [70]	Poland	X				To diagnose the needs of instructors working on the digital inclusion of persons who are excluded, or are at the risk of being excluded, marginalized, or discriminated against in terms of using new technologies
Viktorova et al., 2018 [71]	Ukraine			X		To outline the pedagogical conditions for the successful use of modern ICT in foreign language education for “third age” learners
Wang et al., 2016 [72]	China			X		To examine the barriers that affect the level of educational participation of older adults in China
Wright., 2016 [73]	Walles			X		To learn what people need to learn, what interface changes can reduce cognitive demands, and what to improve in meetings
Zemaitaityte et al., 2018 [74]	Lithuania		X			To review the experience of people arranging studies involving ICT in the University of the Third Age (U3A) organization
Zumarova, 2010 [75]	Czech Republic			X		To contribute to further understanding of senior citizens’ needs in education and ICT utilization since the level of this knowledge represents a limiting factor towards improving the quality of their lives

**Table 2:** Summary of articles focusing on the student (reference, number of people involved in the study, methodology, demographics, and other results).

Reference	N and Methodology	Demographics	Other Results
[63,64]	5561 Survey	Female (0.67)	Barriers
		White (0.95)	Time (0.16)
		Not LGBT (0.97)	Cost (0.07)
		≥ Bachelor’s degree (0.9)	Transport (0.04)
		Not working (0.83)	Physical mobility (0.03)
		<55 yrs. (0.075)	Hearing (0.02)
		55–59 yrs. (0.26)	Health (0.02)
		60–69 yrs. (0.27)	
		70–74 yrs. (0.29)	
		75–79 yrs. (0.17)	
		80–84 yrs. (0.08)	
		85+ yrs. (0.04)	
[58]	256 Survey	Female (0.69)	Beliefs About Aging (0–5)
		Grade 12+ (0.36)	Cognition (2-79)
		Married (0.49)	Agency (0.83)
		60–64 yrs. (0.4)	Interpersonal relationships (2.78)
		65–69 yrs. (0.26)	Persona (2.85)
		70+ yrs. (0.3)	Beliefs (total score) (2.81)
		Does not feel old (0.7)	
		Too old to learn (0.8)	
[60]	94 Survey (Two groups based on the learning approach (face-to-face vs. online))	Female (0.53)	
		Mean age (65.7)	
[71]	310 Survey		Motivations for Learning
			Communication (0.65)
			Personal development (0.25)
			Maintain their proactive attitude (0.1)
			Personal development (0.75)
			Small groups (0.45)
			Ability to use modern ICT in everyday life (0.85)
[68]	374 citizens (50+ years), 5 directors, 5 ICT teachers and 10 participants who have already attended an ICT training in senior universities. Survey		
[72]	21 attendees of U3A courses; 22 no attendees Survey	Female (0.58)	
[59]	135 Survey	Female (0.37)	Use smartphones (0.90)
		≥ Bachelor’s degree (0.08)	Use computers (0.42)
		Not working (0.9)	Use tablets (0.13)
		Married/de facto (0.6)	
		Mean age (74.5)	
		65–69 yrs. (0.27)	
		70–74 yrs. (0.21)	
		75–79 yrs. (0-25)	
		80+ yrs (0.27)	
[15]	13 Non-participant observation	Female (0.54)	Familiar with computer while they were still working (0.8)
		Grade 9+ (0.62)	Discovered Skype or ICQ for communicating (0.24)
		Married (0.92)	
[18]	736 Structured interviews	Mean age (75)	

## Data Availability

The data presented in this study are available on request from the corresponding author.
